# Caliper-Based Restricted Kinematic Alignment Total Knee Arthroplasty

**DOI:** 10.7759/cureus.52780

**Published:** 2024-01-23

**Authors:** Takahiro Okajima, Takafumi Hiranaka, Yasuhiro Fukai, Sho Tanaka, Motoki Koide, Takaaki Fujishiro, Koji Okamoto

**Affiliations:** 1 Department of Orthopaedic Surgery and Joint Surgery Centre, Takatsuki General Hospital, Takatsuki, JPN

**Keywords:** restriction, kinematic alignment, arthroplasty, treatment, knee

## Abstract

Restricted kinematic alignment total knee arthroplasty (rKA-TKA) is a reasonable selection for avoiding an extreme alignment that has been conceded to induce implant failure. However, computer-aided devices (CAS), such as navigation, robotics, and patient-specific instrumentation, are necessary to perform rKA-TKA. This paper reports on the surgical technique of kinematic alignment total knee arthroplasty (KA-TKA) using mechanical instruments. The lateral distal femoral angle (LDFA) and the medial proximal tibial angle (MPTA) are measured from preoperative long radiographs or CT of the lower limb, and the arithmetic hip-knee-ankle angle (aHKA) is calculated from the MPTA - LDFA. The predefined restriction boundaries are used to determine the osteotomy angle. In our practice, the LDFA is 85° to 93°, the MPTA is 85° to 90°, and the aHKA is 5° varus to 3° valgus. If correction of the femoral osteotomy is required, this can be achieved by changing the thickness of the paddle set on the distal articular surface or by adjusting the angle of the variable angle femoral cutting guide. For the tibia, the distal end of the extramedullary rod, with the proximal part placed in the center of the knee joint, should be adjusted so that it does not exceed the lateral malleolus. This limits the medial tilt of the osteotomy plane to within 5.5°. These techniques allow restricted KA to be performed with existing mechanical instruments without using CAS.

## Introduction

Kinematic alignment total knee arthroplasty (KA-TKA) has emerged as a noteworthy development in orthopedic surgery, demonstrating promising long-term outcomes [1]. KA-TKA traditionally involves bone cuts equivalent to the thickness of the component to offset cartilage wear [2]. However, in the presence of bone defects or extreme constitutional alignment, such cuts can lead to excessive alignment, increasing the risk of implant failure due to complications such as loosening [3]. A modified technique, restricted KA-TKA (rKA-TKA), has been introduced to mitigate these risks [4]. This method limits osteotomies to a pre-determined safety zone, though the precise boundaries may vary between studies. This restriction aims to maintain ligament balance and promote favorable clinical outcomes [5].

A significant hurdle in the implementation of rKA-TKA is the reliance on computer-assisted surgery (CAS) methods, including navigation systems, robotics, and patient-specific instrumentation (PSI). These technologies provide precision in controlling bone cut alignment but come with high costs and are not universally available across institutions. Considering these limitations, we propose a novel approach: a calibrated rKA-TKA procedure that employs manual instruments, effectively removing the need for costly and inaccessible CAS technology.

## Technical report

Preoperative planning

**Figure 1:**
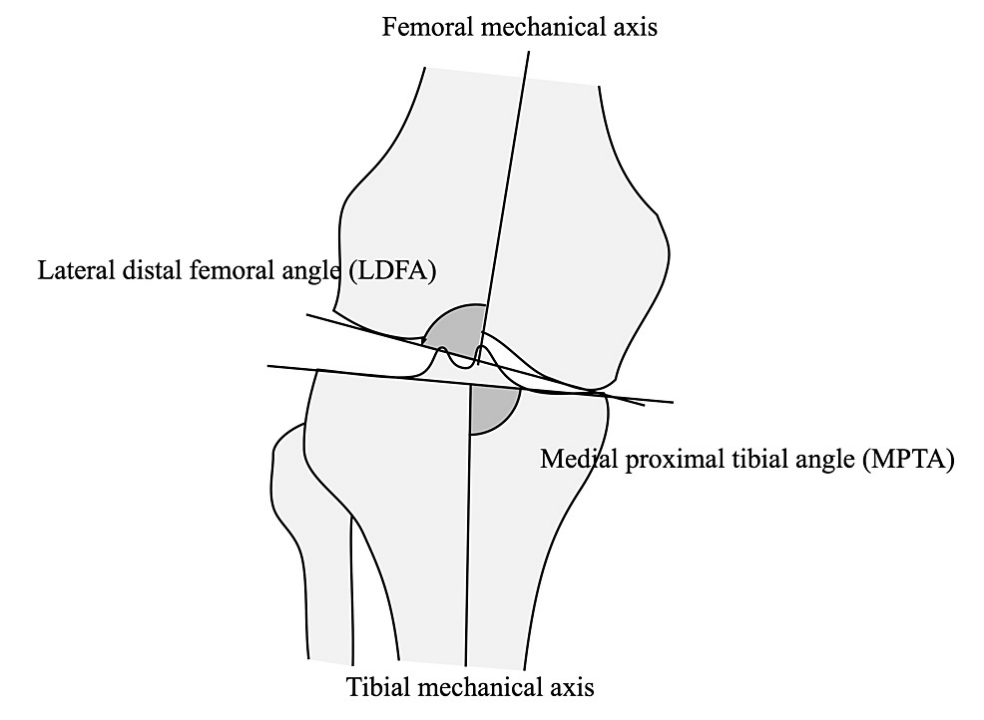
Radiographic measurement On the preoperative long leg radiography or 3D CT, the lateral distal femoral angle (LDFA) and the medial proximal tibial angle (MPTA) are measured as the angle between the mechanical axis and bone surface. The arithmetic hip-knee-ankle angle is calculated as MPTA – LDFA. Image credit: Takafumi Hiranaka

In the preoperative phase, key measurements, such as the lateral distal femoral angle (LDFA) and the medial proximal tibial angle (MPTA), are taken utilizing long leg radiographs or lower extremity computed tomography scans (Figure 1). Subsequently, these values are used to compute the arithmetic hip-knee-ankle angle (aHKA) by subtracting the LDFA from the MPTA [6]. We determine the amount of varus/valgus cuts based on preoperative MPTA and LDFA. Sometimes there is a bone defect in the tibia, so it is important to determine the amount of cuts before the operation. The tibial cut is to be the same as the preoperative MPTA and the femur cut uses a measured technique, which just cuts the amount of implant thickness. These calculations aid in establishing the boundaries of restriction.

Although consensus on this practice is not yet universal, several established protocols exist, which are delineated in Table 1 [4,7,8]. Our approach involves targeting an LDFA between 85° and 93°, an MPTA within the range of 85°-90°, and an aHKA from -5° to +3°. In cases where the LDFA and/or MPTA were corrected within the boundary but HKA is still out of the boundary, the LDFA is adjusted to below 90°. We applied this protocol for all patients, and whether the restriction was needed or not was decided preoperatively.

**Table 1:** Target of range of HKA, LDFA, and MPTA in prior studies HKA: hip-knee-ankle angle; LDFA: lateral distal femoral angle; MPTA: medial proximal tibial angle

	HKA	LDFA	MPTA
Almaawai (2017)	-3 to 3°	85 to 95°	85 to 95°
McEwen (2018)	-6 to 3°	84 to 93°	84 to 93°
MacDessi (2020)	-5 to 4°	86 to 93°	86 to 93°

Surgical technique

**Figure 2:**
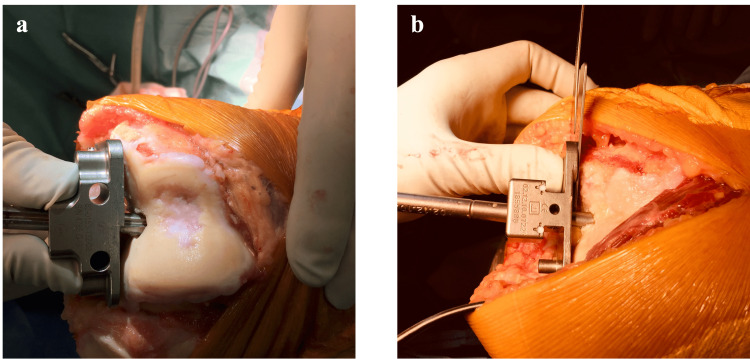
Manipulation of the distal femoral cutting plane using a paddle a. A paddle for the kinematic alignment total knee arthroplasty is inserted through the intramedullary rod, and both planes are paced at the distal femoral condyles. There are 2 mm differences between the plane of the paddle, compensation for the cartilage defect. The cutting plane is expected to be parallel to the bone surface. b. If a correction of the cutting plane is required, a defined thickness of metal plate, such as an angel wing and a shim, is interposed between the articular surface and the paddle plane. A 1 mm additional thickness is equivalent to about 1° corretion.

A paddle is utilized to create a bony foramen in the distal center of the femur. An intramedullary rod is subsequently inserted into this opening. The paddle is then positioned against the distal femoral condyle. If the condyle's cartilage remains intact, the paddle is set to execute the osteotomy 9 mm away from the cartilage surface. If there is a cartilage defect, the osteotomy is conducted 7 mm from the surface with a 2 mm correction (Figure 2a) [2].

In the majority of cases, no femoral osteotomy adjustment is required. However, certain scenarios, such as a discernible femoral defect or excessively extreme original alignment, may necessitate an adjustment in the osteotomy angle. A metal plate is inserted between the paddle and the articular surface to make this adjustment. If a decrease in the LDFA (to be valgus) is needed, the metal plate is inserted on the medial condyle side (Figure 2b). Conversely, if an increase (to be varus) is required, it is placed on the lateral condyle side. Angle adjustment is made assuming that a 1 mm insertion modifies the angle by approximately 1° based on our previous study (personal data). This metal plate can be an angled wing or saw blade.

When using the Persona(r) system, the incremental thickness of shims utilized for bearing trials also serves this purpose. Following these steps, the cutting block is attached, and the osteotomy is conducted. The osteotomy's extent is then measured with calipers, considering the blade's thickness and the added metal plate's thickness.

**Figure 3:**
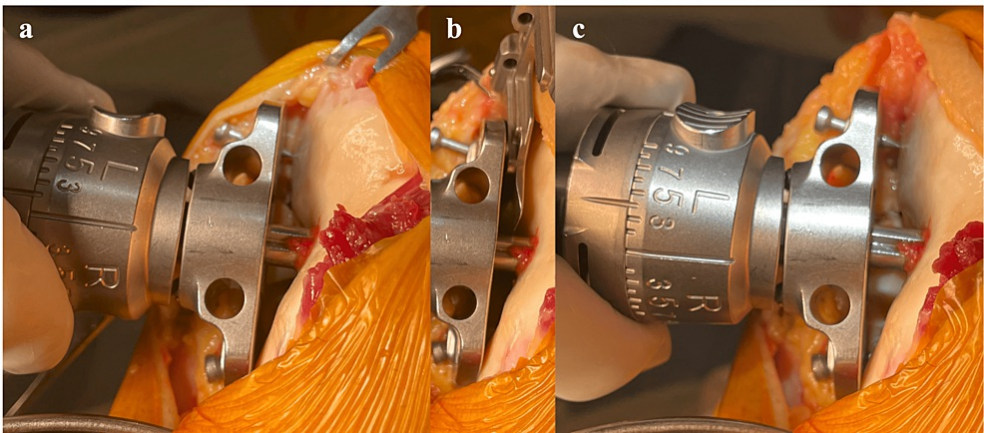
Distal femoral cutting adjustment using Persona (TM) dial femoral guide a. The cutting angle is adjusted so that there is a 2 mm space between the paddle plane and bone surface, and the scale was read at this time. b. The space can be verified using a 2 mm metal plate such as a shim. c. LDFA can be adjusted based on the previously recorded angle scale, For instance, the cutting plane is planned to be 2° in more valgus, and the dial is adjusted to + 2°. LDFA: lateral distal femoral angle

When employing the Persona™ system, a dial osteotomy guide is used to manage the osteotomy [9]. Initially, an intramedullary rod is inserted, as described in the previous section. Subsequently, a dial osteotomy guide is positioned, and a 2 mm metal plate to compensate for the cartilage defect is laid over the condyle where the cartilage wear is observed (Figure 3a). The measurements from the cutting guide are then recorded when the paddles on either side of the guide make contact with the articular surface or the metal plate. Based on these readings, the cutting guide is adjusted to varus or valgus as required (Figure 3b).

Following the osteotomy, the rod is reinserted, and the guide that is set as the desired alignment is applied to verify that the osteotomy has been executed as per the plan (Figure 3c). In this method, avoiding excessive widening of the bony foramen where the intramedullary rod is inserted is critical. Excessive widening could allow the rod to move within the foramen, thus compromising alignment accuracy.

Posterior femoral condyle osteotomy

**Figure 4:**
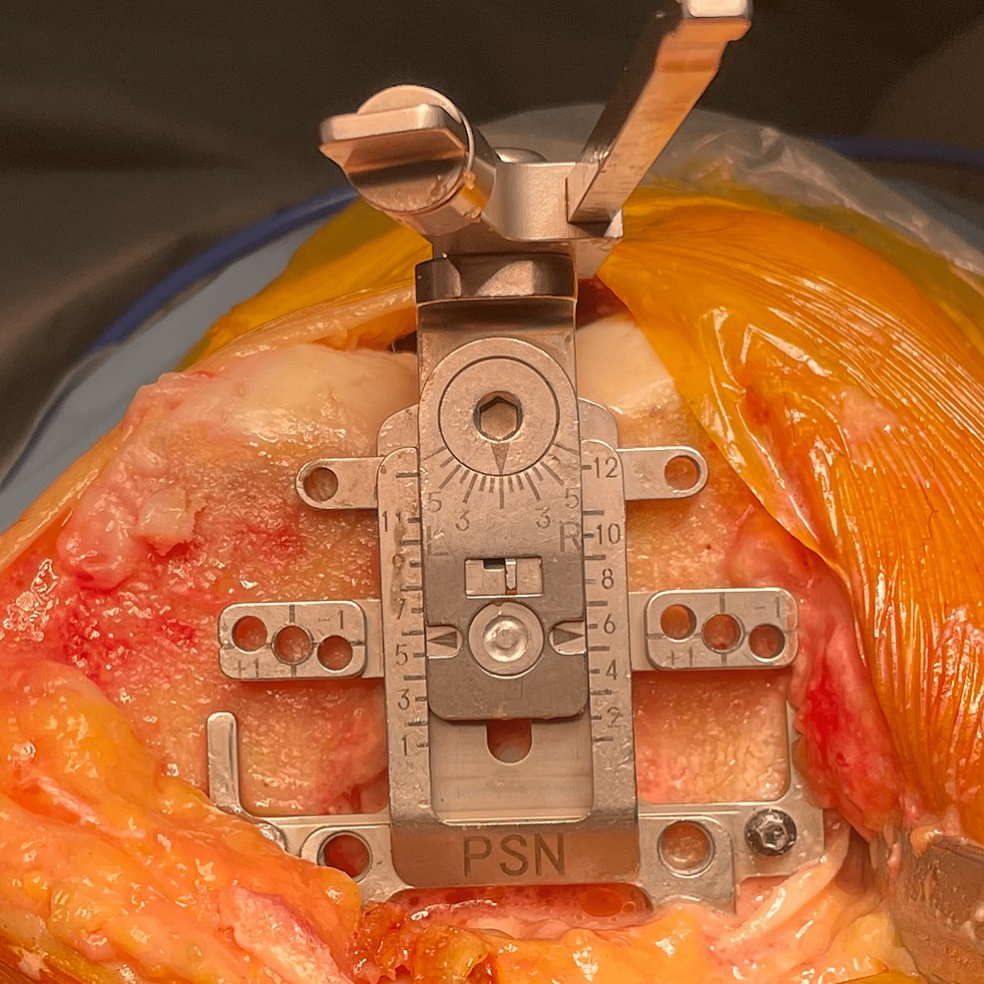
The definition of femoral rotation and size We prefer to use an anterior reference sizer. After confirmation that both posterior condyle cartilages are intact, the rotation angle is set as 0°. If the scale indicates between the scales, the sizer is adjusted so that the scale indicates the bigger size.

The osteotomy of the posterior femoral condyle should be equivalent to the component thickness on both sides, assuming no cartilage defect at the posterior condyle; then the rotation angle of the femoral sizer is 0° (Figure 4). Most component posterior condyles range from 8 mm to 9 mm in thickness. If a cartilage defect exists, the external rotation angle should be adjusted to accommodate this defect or a metal plate should be inserted into the paddle on the side where cartilage wear is observed. The rotation angle should be adjusted by 1° for each mm of cartilage defect.

First, an osteotomy of the posterior condyle is conducted, and the bone thickness is measured. If the observed error is 2 mm or more, a posteriorly referenced sizer is typically used to recut by adjusting the rotation of the four-sided cutting block. However, we advocate for using an anterior reference. With an anterior reference, even if the size measurement exceeds the midpoint of the guide, the thickness of the posterior condyle can be maintained constant (as with the posterior reference guide) by adjusting to the larger size. Suppose the posterior bone cut is not as planned, and a recut is needed. In that case, the size can be adjusted to alter the amount of posterior osteotomy without affecting the anterior osteotomy surface.

**Figure 5:**
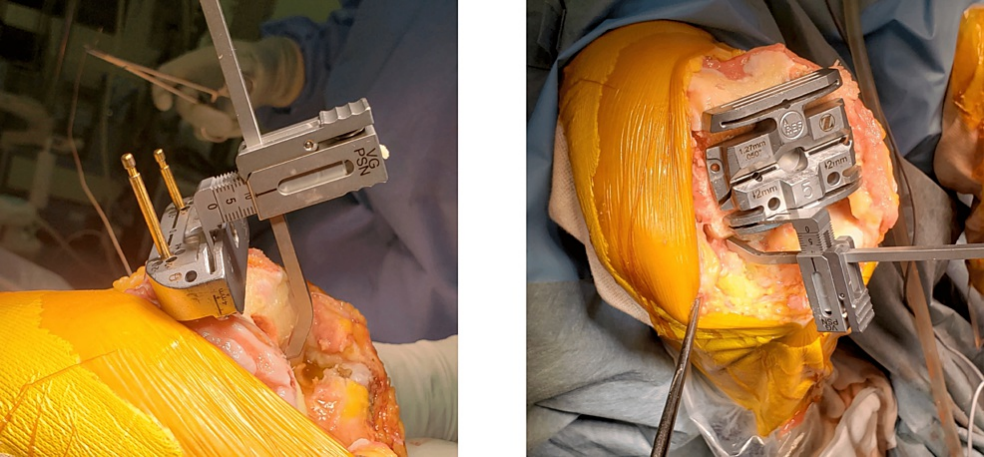
Verification of bone-cutting thickness Using an adjustable stylus, bone cut thicknesses are evaluated. If the tip of the stylus just touches on bone or cartilage surface, the position of the cutting block is considered to be proper.

If the adjustable stylus is available, the accuracy of cutting block placement is confirmed by the styles, which is set as the component thickness and inserted in the cutting slot; then the tip of the stylus should be just on the bone surface (Figure 5).

Tibial osteotomy

The fundamental procedure of the tibial osteotomy is carried out parallel to the tibial articular surface. Initially, an extramedullary tibial guide is positioned, aligning the center of the proximal guide with the midpoint of the tibial intercondylar eminences. Subsequently, the posterior tilt is adjusted [9].

**Figure 6:**
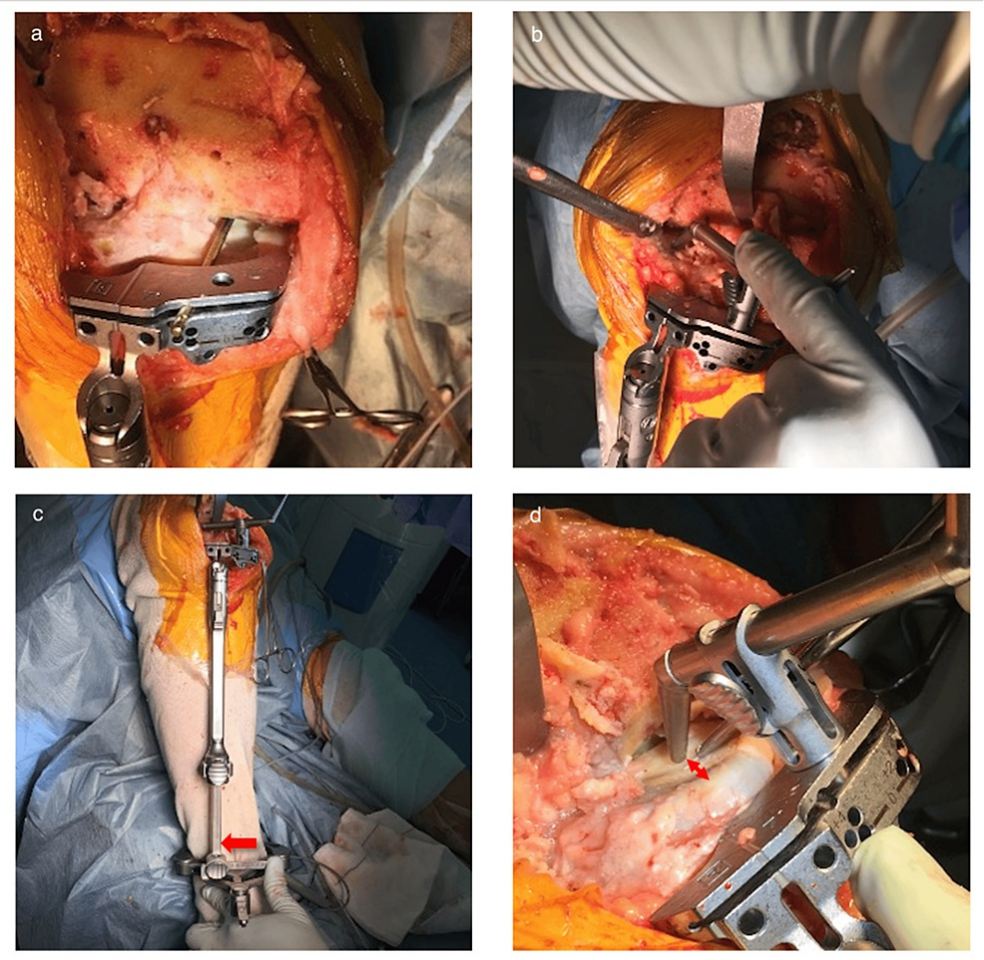
Procedure of tibial osteotomy a. A pin or angel wing penetrating the slot of the tibial cutting block is adjusted to be parallel to the medial tibial plateau. b. Marinating the posterior slope, a 10 mm stylus is set at the center of the lateral tibial plateau. c. The ankle piece of the extramedullary rod is shifted laterally (red arrow) but never exceeds the lateral malleolus to restrict the varus angle of the tibial cutting surface up to 5 to 6 degrees. d. The inclination of the extramedullary rod is adjusted so that there is a 2 mm space between the tip of the 10 mm stylus and the medial tibial bone surface (red arrow).

The angled wing is placed on the upper surface of the tibial osteotomy block or a pin is inserted through the hole in the Persona cutting block. The sloping angled wing or pin is positioned on the medial femoral plateau to run parallel to the articular surface (Figure 6a). The posterior tilt is fine-tuned to achieve parallel alignment with the joint plane. Considering the preconfigured posterior tilt angle of the cutting block, the posterior tilt can be slightly reduced if it is anticipated to exceed 10 degrees. The posterior slope is aligned with the medial plateau. If there is a medial bone defect, it is aligned to the lateral. If the lateral plateau also has a bone defect, the posterior tilt is determined to be 7°.

Following this, a 10-mm stylus is positioned at the lateral plateau (Figure 6b), and the distal end of the extramedullary guide is moved laterally to internalize the osteotomy surface while preserving its height (Figure 6c). The osteotomy thickness on the medial plateau is then fine-tuned to 8 mm, referring to the residual cartilage margin. Another stylus with 8 mm is available. The simultaneous use of 10 mm lateral and 8 mm medial styluses makes it easy to decide the inclination. However, even with a 10-mm stylus, one can verify the 8-mm measurement by calculating the gap between the stylus tip and the cartilage margin using the tip of an electrocautery scalpel or similar tool (Figure 6d).

Suppose the distal end of the extramedullary rod deviates beyond the lateral malleolus. In that case, the tilt should be decreased so that the distal end of the extramedullary rod comes anteriorly to the lateral malleolus. This adjustment ensures that the medial tilt of the osteotomy plane is kept within approximately 5.5° [10].

Implant trial 

After the bone cuts, bone gaps are evaluated by spacer block. We allow a slightly looser lateral gap, but if the imbalance is extreme, a tibial recut is considered. 

## Discussion

The most significant feature of this report is that it is possible to perform rKA using conventional surgical instruments without the need for computer-assisted surgery (CAS). While there are numerous reports on rKA [4,5,11], they all involve using robots or patient-specific instrumentation (PSI). There are no reports of rKA using conventional manual instruments. This is the first report of rKA using them.

In Asian countries, constitutional varus is considered to be prevalent [12]. MacDessi et al. reported that only half of the Western population required adjustments [4] while Suda et al. [13] found that when existing boundaries were used for adjustments in Japan, about 80% needed adjustments. Without boundary restrictions in KA, most cases would have excessive varus tibia competent placement and leg alignment. Recent reports have shown that the medium and long-term results were favorable even with unrestricted KA [1,14]. However, these reports are based on Western populations, and it may be risky to apply the results directly to Asians with different anatomy because reports on unrestricted KA from Asia are still limited. Therefore, it might be safe to impose restrictions and then reassess whether to continue or release them as the evidence grows.

In this technique, a method of alignment restriction in the femur has been presented. Many past reports on KA suggest that distal femoral bone defects can be ignored [15,16], and compensation for cartilage thickness has been uniformly set at 2 mm. However, apparent bone defects are occasionally seen when observing the femoral joint surface. There are cases where the lateral distal femoral angle (LDFA) shows significantly higher values than the opposite side, suggesting bone defects. Although there is currently no reliable method to infer the amount of femoral bone defect, limiting the LDFA to about 93° for cases with obvious bone defects is better. In any case, it is necessary to estimate femoral bone defects and determine appropriate LDFA boundaries in the future.

**Figure 7:**
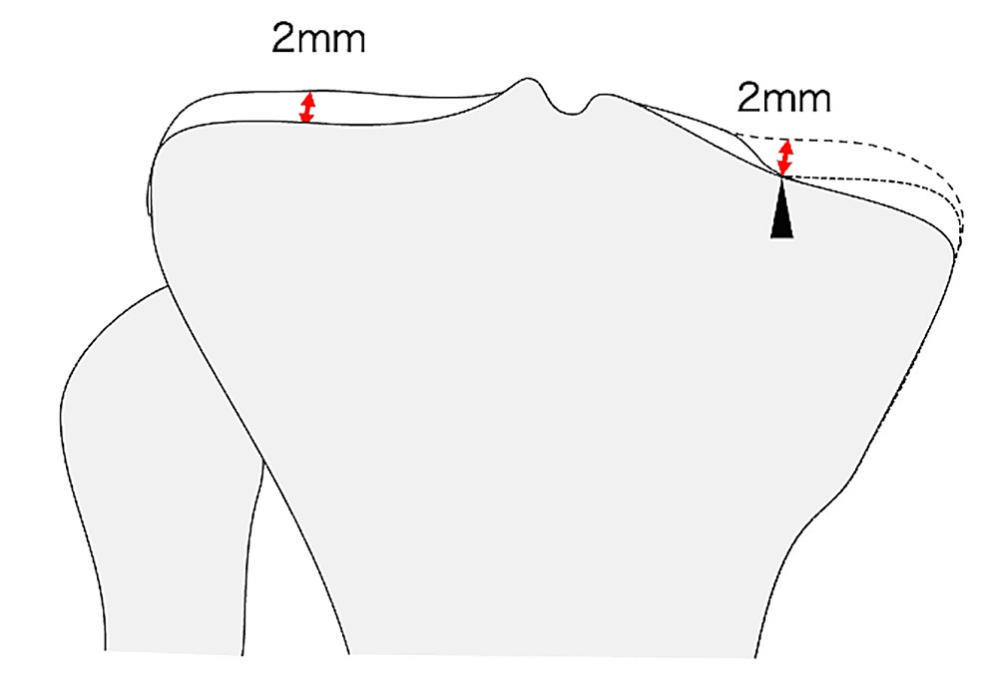
The reference point for the medial stylus The medial margin of the remnant cartilage near the base of the medial tibial spine (black arrowhead) is the reference point of the medial stylus. The point should be 2 mm (normal cartilage thickness) beneath the pre-arthritic articular surface, even in knees with a bone defect. Image credit: Takafumi Hiranaka

Regarding the tibia, it is also possible to restrict the varus to no more than 5.5° by installing the tibial cutting guide parallel to the joint surface first and then positioning the distal end of the extramedullary rod so as not to exceed the lateral malleolus [10]. It has been reported that using this method, most cases could be limited to within 5° and almost all cases to within 6°. Therefore, stable restrictions are possible. However, it is difficult to evaluate the joint surface of the affected tibial plateau. Some cases have bone defects, so when the medial plateau is set as a reference point of the bone cut, it is necessary to compensate for not only cartilage but also bony defects. The previous reports used the medial and lateral intercondylar eminence bases as reference points [15]. Still, they are not always suitable because they may already have cartilage defects or be lowered from the joint surface. We used the residual cartilage margin as a reference point. This part has no bone defects and can be considered a point 2 mm below the cartilage (Figure 7). This is also considered useful in unrestricted KA.

Limitations

There are limitations to this technique. First, the purpose of this report was to describe the details of the technique, so no results have been shown. It is necessary to verify the results of using this technique. The second is that there is no method to estimate femoral bone defects. This applies not only to this technique but to all KA techniques. It is impossible to accurately reproduce the joint surface without evaluating the presence or absence of bone defects and, if present, how much. Third, we have set our restriction boundaries to DFA 85°-93°, MPTA 85°-90°, and aHKA -5° - + 3°, but we consider these as temporary. This is the same for the boundaries in other reports. In the future, we believe it is necessary to set evidence-based boundaries from a large amount of data. Furthermore, these may vary based on race, gender, and age. Fourth, this technique does not use robots. Robotics can provide more accurate bone cuts, but it needs a higher cost to introduce and maintain it, so it has not been used in most institutions. Therefore, the mechanical-based technique is still beneficial for most surgeons.

## Conclusions

In conclusion, this report underscores the feasibility of performing rKA using conventional surgical instruments without CAS. In the future, it will be necessary to verify whether rKA is accurately performed using this method, as well as to establish a method for evaluating bone defects on the femoral side and to set the boundary based on evidence.
